# Edemas of the face and lymphoscintigraphic examination

**DOI:** 10.1038/s41598-021-85835-w

**Published:** 2021-03-19

**Authors:** Pierre Bourgeois, E. Peters, A. Van Mieghem, A. Vrancken, G. Giacalone, A. Zeltzer

**Affiliations:** 1 Service of Dermatology, Hospital Erasme, Université Libre de Bruxelles, 808, Route de Lennik, 1070 Brussels, Belgium; 2 Services of Nuclear Medicine, Institute Jules Bordet and HIS-IZZ, Université Libre de Bruxelles, 1, rue Héger-Bordet, 1000 Brussels, Belgium; 3 Multi-Disciplinary Clinic of Lymphology, Institute Jules Bordet and Hospital Erasme, HIS-IZZ Hospital, Université Libre de Bruxelles, 63, rue Jean Paquot, 1050 Brussels, Belgium; 4 Department of Lymphatic Surgery, AZ Sint-Maarten, Liersesteenweh, 435, 2800 Mechelen, Belgium; 5 Department of Nuclear Medicine, AZ Sint-Maarten, 435, 2800 LiersesteenwehMechelen, Belgium; 6 Department of Plastic and Reconstructive Surgery, European Center for Lymphedema Surgery, Universitair Ziekenhuis Brussel, Vrije Universiteit Brussel, 101, Laarbeeklaan, 1090 Brussels, Belgium

**Keywords:** Medical research, Pathogenesis

## Abstract

Facial edemas not secondary to surgery and/or radiotherapy for head and neck cancer are relatively uncommon. Our aim is to report a retrospective analysis of the lymphoscintigraphic and SPECT-CT investigations obtained in patients with such facial edema. Retrospective review of exams (planar imagings in all and with SPECT-CT in 5) obtained after the subcutaneous injection of 99mTc HSA Nanosized colloids between the eyebrows in five men and seven women. Four main lymphatic pathways were identified on sequential planar imagings: para-nasal left and right and supra- ocular left and right. For eleven patients, the absence of visualization of lymphatic drainage and/or their delayed appearance correlated well with the localisation of the edematous areas. In two patients with post-traumatic and post- surgical edemas, SPECT-CT showed one deep left sided cervical lymph node (LN) in front of the first cervical vertebra. This lymphoscintigraphic approach represents a simple and valuable way to assess the lymphatic drainage pathways of the face and to establish the diagnosis of facial lymphedema.

## Introduction

In the literature, attention has mainly been given to lymphedemas and other edemas of lymphatic origin involving the extremities^1,2^ for which lymphoscintigraphy is considered as the gold standard for evaluation^3,4^. Head and Neck (HN) Lymphedemas (HNLE) are less frequent. Their incidence, when secondary to treatment for head and neck cancer, is however reported to vary between 12 and 54% of all patients^5^. HN edemas not related to cancer treatment have also been described in the medical literature for 70 years by now^6–8^. They may be benign and/or associated to syndrome and/or genetic diseases^9–15^. Their medical work-up lead to the use of CT, MRI, Ultra-Sound, angiography, PET-CT, lympho-scintigraphic and lympho-fluoroscopic investigations of the head and sometimes require biopsy of the skin, genetic testing and investigations of other organs^15,16^. To our knowledge, the lymphoscintigraphic investigations of the face in such cases have been reported in only four cases^16^ and in one case report^17^.

The aim of this brief communication is to report our experience and results obtained in patients with facial edema non-secondary to cancer with a simple lymphoscintigraphic approach.

## Material and methods

This is a retrospective observational review of exam performed from 2005 to 2019 in patients who came for the investigation of one edema (limited or not) at the level of the face. Lymphoscintigraphy was performed to evaluate the lymphatic drainages for diagnostic and/or therapeutic purposes after patient’s informed consent (as for any other exam proposed and performed for any patient). All medical data and information regarding the patients included in this study were used in agreement with the rules of conduct dictated by our institution and in agreement with and with the approval by the Ethics Committee of the Jules Bordet Institute (Ethics Committee document number 2048).

Patients characteristics are given in Table 1.

**Table 1:** Characteristics of the investigated patients with the etiological diagnosis of their edema at the level of the face.

No	Sex	Age	Diagnosis
1	F	55	1ary
2	M	30	1ary congenital (Mucoviscidosis)
3	F	47	1ary
4	M	45	Post-trauma
5	F	44	Yellow Nail syndrom
6	F	65	Post-Botox
7	M	42	Morbihan Syndrom
8	M	23	Post-trauma
9	M	29	No definitive etiological diagnosis (1ary?)
10	F	21	1ary congenital microcystic lymphangiomas
11	F	70	Post-trauma
12	F	49	After facial lifting

*No.* patient number, *F* female, *M* male.

Planar scintigraphic images were obtained after the subcutaneous injection of 0.2 ml of 99mTc-labeled (81 MBq) HSA nano-sized colloids (Nanocoll, GE Healthcare, Little Chalfont, United Kingdom, one tenth of one vial) at the level of the forehead between the eyebrows, just after the injection, after pinching and stretching the injection site, after a massage of the injected site and after manual lymphatic drainage of the injection site (see Figs. 2 and 3). When available, Single-Photon Emission Computed Tomography (SPECT) combined with a X-ray Computed Tomography (CT) (or SPECT-CT) was also obtained in five (patients n° 4, 8, 10, 11 and 12) with the same dual-headed gamma-camera system.

### Ethics approval

Retrospective monocentric review of materials approved by the institutional review board ethics committee.

## Results

**Figure 1:**
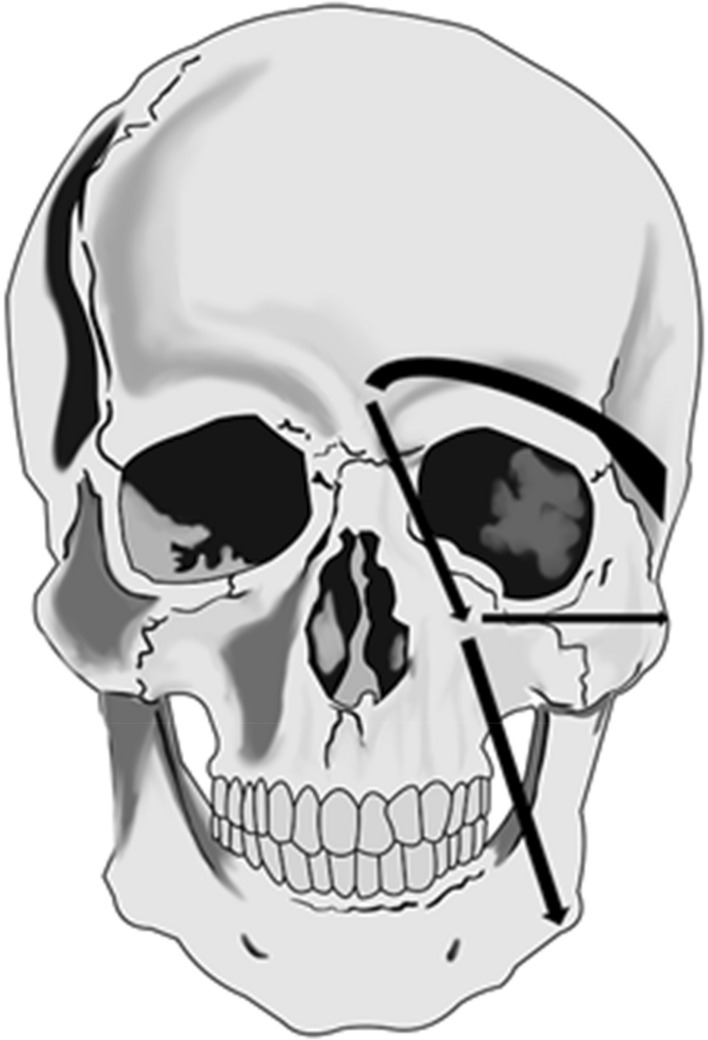
Anterior view of the face with arrows showing (on the left side) the lymphatic drainages of the injected site: supra-ocular to the pre-auricular LN and para-nasal to (1) the sub-mandibular LN, (2) the pre-auricular LN and (3) the parotid LN.

**Figure 2:**
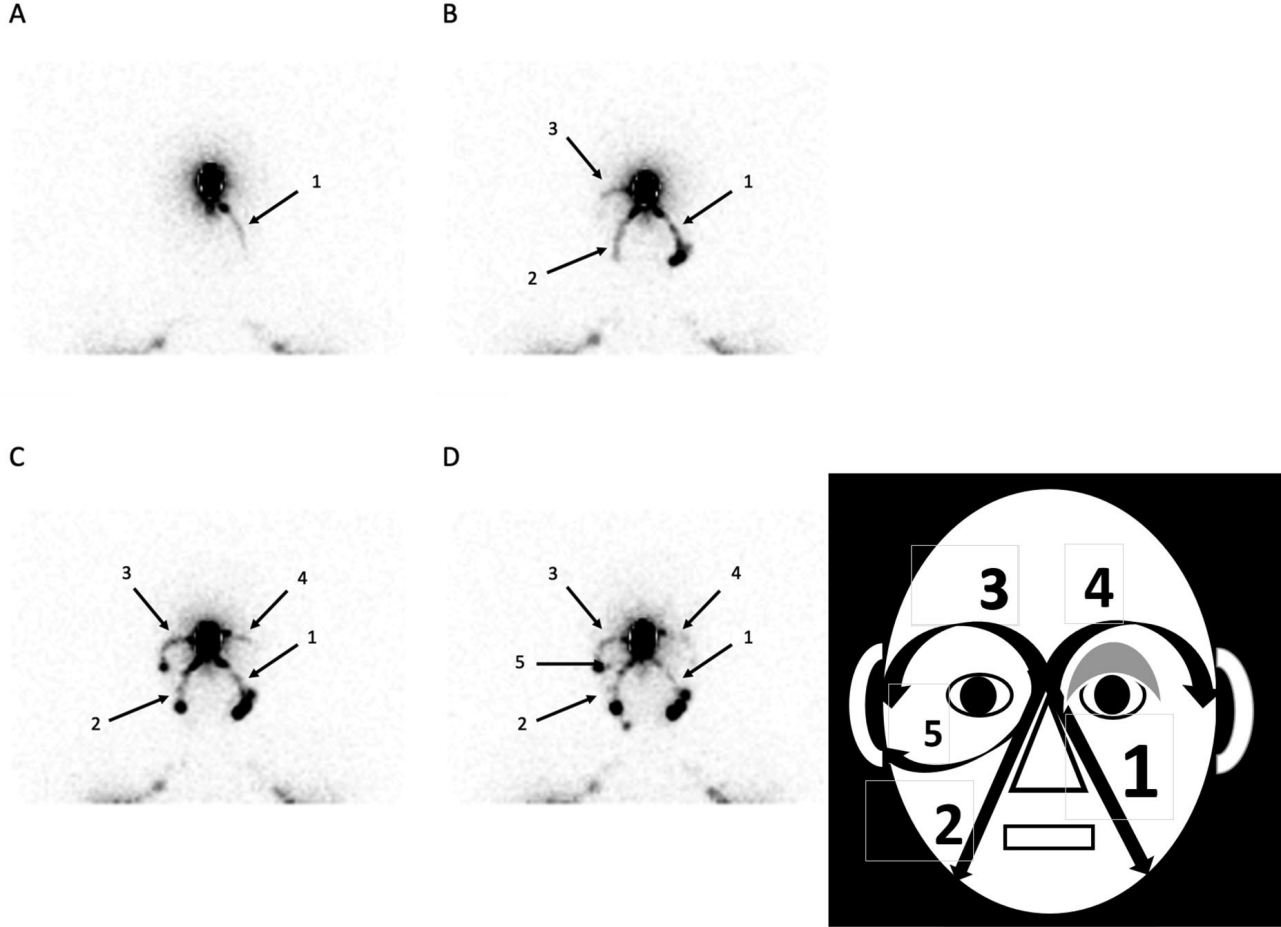
This patient came with an edema at the level of the left eyelid reported as present since birth. His medical history revealed familial genetic disease (mucoviscidosis) and a malformation of the ductus thoracicus on his bipedal lymphoscintigraphic exam (no lower limb edema). His exam shows (see picture on the right and the scintigraphic images on the left) the orderly appearance of the left paranasal drainage (picture **A**, arrows 1), then of the right one (picture **B**, arrows 2) with the right supra-ocular one (picture **B**, arrows 3), of the left supra-ocular one (picture **C**, arrows 4). At the end of the exam (picture **D**), the right infra-ocular lymphatic drainage may be seen internal to the right preauricular lymph node (arrow 5).

Four main lymphatic pathways were identified (see Fig. 1): one para-nasal left and one right (reaching the sub-mandibular LN as first tier LN) and one supra-ocular left and one right (reaching the pre-auricular LN as first tier LN). Infra-ocular lymphatic drainages (see Fig. 2) could also be observed in 2 cases.

**Figure 3:**
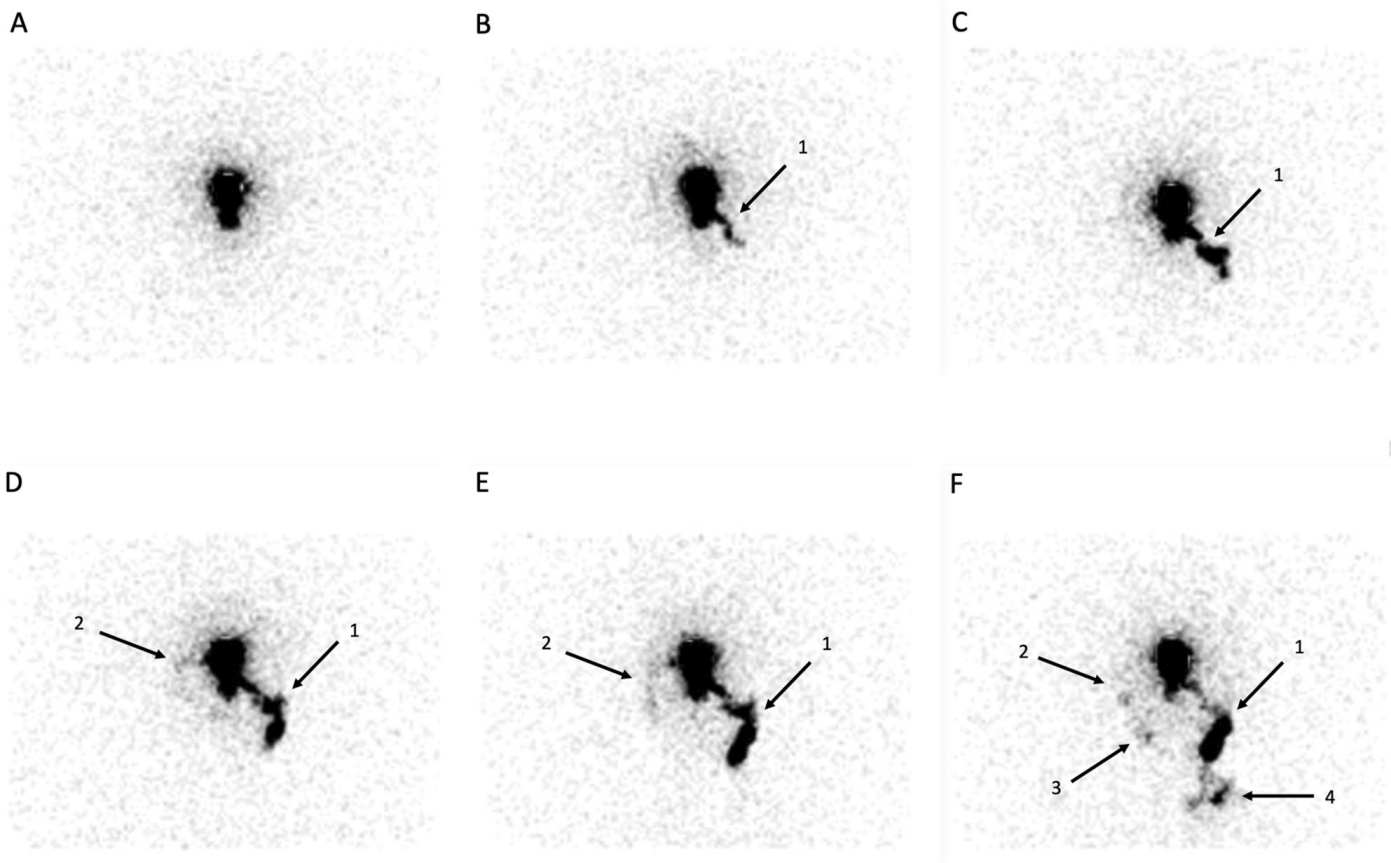
In this case, the patient (after fractures -operated- of the left nasal, zygomatic and supra ocular bones) complained of edema at the level of the face, of enlarged left cervical nodes and of left sinusal edemas. In this case, the lymphatic drainage is quite only and predominantly seen paranasal left sided (see arrows 1) reaching left cervical lymph nodes and finally supraclavicular ones (arrow 4) with, in a 2nd time (see picture **D**,**E**), the appearance of the right supra-ocular drainage (arrows 2) reaching the right pre-auricular LN and -may be- in a third time, of a right para nasal lymphatic drainage reaching a infra-sub-mandibular LN (arrow 3).

**Figure 4:**
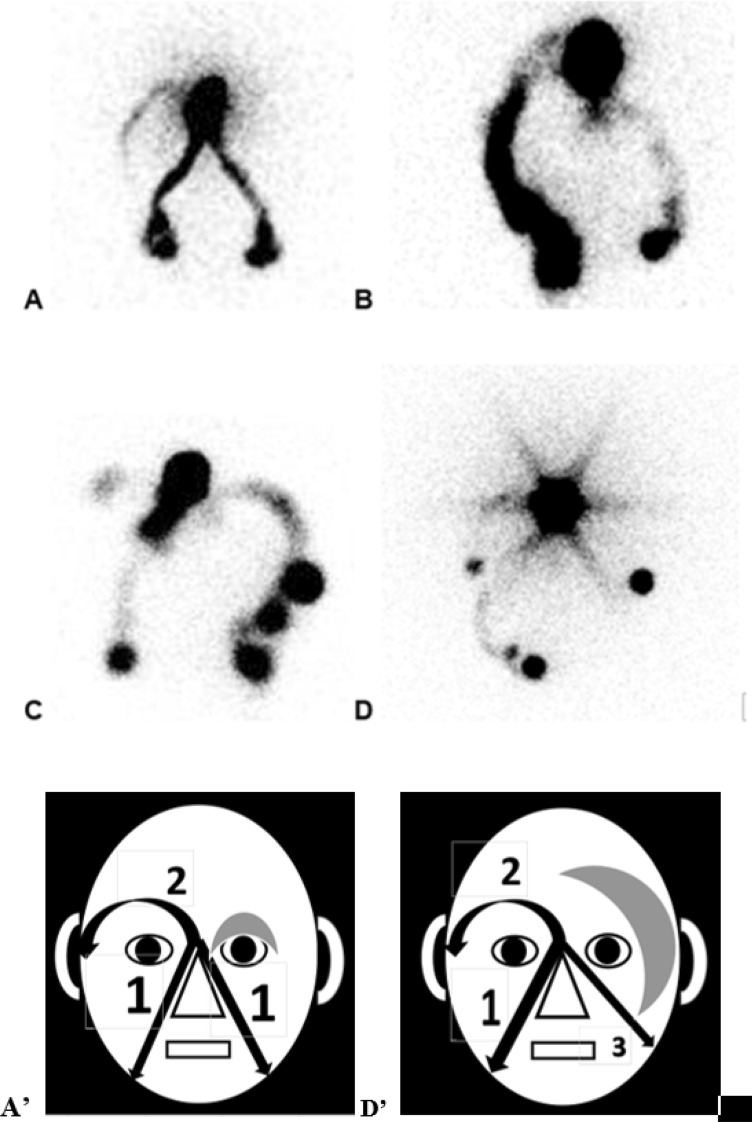
Picture (**A**) (scintigraphic view) and (**D’**) (schematic presentation of the result): in this case referred for isolated edema at the level of the left eyelid (gray zone), the paranasal lymphatic drainages appear normally (arrows 1) while the appearance of the supra-ocular LV is delayed (arrow 2) and while the left supra-ocular lymphatic drainage could not be demonstrated. The 1ary origin of this abnormality was suggested by the demonstration of one associated malformation of the ductus thoracicus by a bipedal lymphoscintigraphy. Picture (**B**) In this case, the patient had injections of Botulinum Toxine at the level of her face. She complained of edema at the level of the eyelids but more pronounced on the left in the upper part and on the right in the external part. In her case, only the right supra ocular and left para nasal drainages could be demonstrated. Picture (**C**) In this patient who was referred with edema at the level of the two eyelids but more pronounced at the inferior part of the right one and who had presented right otitis, the left paranasal lymphatic drainage is not seen, the right supra ocular is not complete and the right para nasal drainage is unusually hyperactive in its upper and apical part. Picture (**D**) (scintigraphic view) and (**D’**) (schematic presentation of the result): this patient had congenital micro-cystic lymphangiomatous lesions at the level of her left edematous hemiface (gray zone). The exam showed normal right para nasal lymphatic drainage (arrow 1) and delayed supra-ocular lymphatic drainage (arrow 2) but no supra ocular on the left side and one abnormal left infra ocular drainage reaching intra-parotid LN (arrow 3).

At least one of these main drainages was not seen (in 7/12: see Figs. 3 and 4) and/or had minor drainage (in 3/12: see Fig. 2) and/or appeared delayed (in 3/12: see Fig. 3). In patients’ number 2, 4, 5, 9 and 12 (see Table 2 and Fig. 2), the 4 main lymphatic pathways could be seen but they did not appear simultaneously.

**Table 2:** Localisation of the edema(s) in the patients and corresponding visualisation of the 4 main lymphatic drainage pathways, either seen or not (with their order of appearance: 1st = first seen: 2nd = second drainage seen:…).

No	Edema(s) localisation	Supra ocular right	Para nasal right	Para nasal left	Supra ocular left	See figures
1	Eyelid left	2nd	1st	1st	Not seen	Figure 4A
2	Eyelid left	3rd	2nd	1st	4th	
3	Eyelids (R > = L)	3rd	1st	1st (< R)	Not seen	
4	Zygomas R > L	4th	2nd (abnormal)	3rd	1st	
5	Eyelids R > L Nose L > R	3rd	1st	4th	2nd	Figure 2
6	Eyelids Upper left and external right	Normal	Not seen	Normal	Not seen	Figure 4B
7	Lower eyelids Right > > left	Stop pre-auricular	Inflammatory	Not seen	Normal	Figure 4C
8	Hemiface left	1st	2nd (< 1)	Not seen	3rd	
9	Hemiface right	4th	2nd	1st	3rd	
10	Hemiface left	3rd	1st	2nd (abnormal)	Not seen	Figure 4D
11	Hemiface left	2nd	2nd	1st	Not seen	Figure 3
12	Hemiface left	2nd	1st	1st (< R)	3rd	

*No.* number.

For eleven patients, the absence of visualization of lymphatic drainage and/or their delayed appearance correlated well with and explained the localization of the edematous areas (see Table 2). The situation for patient n° 11 might be considered as “paradoxical” but will be discussed further (see Fig. 3).

Interestingly, in two patients with post-traumatic and post- surgical edemas (of the three patients with SPECT-CT exam), abnormal lymphatic drainage of the face was observed and the SPECT-CT showed one left sided deep cervical LN in front of the FIrst cervical vertebra.

## Discussion

Except one case report in one child with cystic hygroma^17^, only a single reference was found in the medical literature about the use of lymphoscintigraphy to evaluate and assess the lymphatic lesions in patients with edema of benign origin at the level of the face. Nittner-Marszalska et al.^16^ reported four cases with one (unilateral) or two (bilateral) injections of radiocolloids at the level of the upper lip. In the four patients, they observed no drainage towards the sub-mandibular lymph nodes but their restoration in 3 on follow-up exam corresponded with clinical improvement of the edema.

With a single injection between the eyebrows as proposed here, we can demonstrate not only the four main major lymphatic drainage pathways of the face but also their “absence” and/or the sequence of their appearance. The “absence” of visualization of these pathways has to be well understood: these lymphatic vessels may be not present at all due to agenesis (in case of primary lymphangio-aplasia) and/or could be obstructed (e.g. in lymphangio-obliteration either primary, or secondary to lymphangitis and/or the result of prolonged tissue inflammation).

In eleven out of twelve patients, we show that such simple subcutaneous injection of 99mTc labelled HSA nanosized colloids followed by planar imagings enabled to demonstrate the “absence” and/or the relative functional decrease-impairment of one or more superficial lymphatic drainages which explains the edemas clinically observed. In two of the five patients who also benefited from a SPECT-CT acquisition, the exam also showed deep drainage that could not be visualized on planar imagings.

In one patient (no. 11), the result might appear somewhat paradoxical (when compared to what was found in other patients) with an increased superficial lymphatic flow in the left paranasal area, the side of which the patient complained. However, this specific situation might be explained by the fact that, with symptoms of left sinusal edemas, the lymphatic problem (impaired lymphatic drainage) might rather be deep than superficial and, to put it in another way, that the deep lymphatic vessels remain obliterated and/or hypo-functional after the trauma and that this increased superficial drainage may represent a compensatory situation to one increased lymph load. In case of lower limb edemas, such increased “paradoxal” superficial lymphatic drainage were recently demonstrated as a compensatory phenomenon to deep lymphatic problems^18^.

Physical treatments (compression therapy and/or manual lymph drainage) have been reported to be useful in patients with edema of the head and neck secondary to cancer surgery and/or radiotherapy^19,20^. However, Deng et al.^21^ suggested that their potential benefit was underscored due to substantial barriers to their application (patients’ participation and compliance). They concluded that personalized strategies should be considered for ensuring optimal patient outcomes. The use of lymphoscintigraphy could therefore represent one way to improve the results of the manual lymph drainage approaches and the patients’ compliance to these treatments.

Lymphofluoroscopy or Near Infra Red Fluorescence Imaging (NIRFI) of the lymphatic vessels after the injection of Indocyanine Green (ICG) may represent in such cases a non-radio-active, non-irradiating alternative technique to lymphoscintigraphy. It was proposed by Maus et al. in 2010 for edema of the face^22^ and is used by surgeons who are performing lymphatico-venous anastomosis^23^. However and until now, ICG is only approved for the imaging of the Sentinel Lymph Node in Cervix and Corpus Uteri cancers (ref HCPCS Code **C9756**) and there are concerns about its potential toxicity on the lymphatic vessels^24,25^. The temporary tattooing of the injected site may also represent one drawback for some patients^26^. More detailed than lymphoscintigraphy, lympho-fluoroscopy is able to visualize some lymphatic pathways (infra-orbital for instance) and/or dermal backflows phenomenoma (if present) better than the radionuclide approach. However, due to its physical limitations (only superficial fluorescent structures can be seen), the demonstration of the deep lymph nodes (allowed by lympho-SPECT-CT) would have not been possible in our two patients with post-traumatic edema of the face.

## Conclusion

The lymphoscintigraphic investigation of the face after injection of 99mTc labelled colloids at the level of the forehead between the eyebrows appears to represent a simple and valuable way to assess the lymphatic drainage pathways of the face and to establish the diagnosis of facial lymphedema. SPECT-CT acquisition is also useful in patients with post-traumatic edemas to demonstrate deep lymphatic drainages not seen on planar imaging.
